# Excimer light versus excimer light plus trichloroacetic acid for vitiligo treatment: A comparative study

**DOI:** 10.1097/MD.0000000000041774

**Published:** 2025-03-07

**Authors:** Salman Bin Dayel, Refaat R. M. Hammad, Ramadan S. Hussein, Shaimaa E. A. Badawy, Othman Abahussein, Abeer Ali El-Sherbiny, Mofreh Mansour

**Affiliations:** a Department of Dermatology, College of Medicine, Prince Sattam Bin Abdulaziz University, Al-Kharj, Saudi Arabia; b Department of Dermatology, Andrology and Venereology, Faculty of Medicine, Al-Azhar University, Cairo, Egypt; c Department of Medical Laboratory, College of Applied Medical Sciences, Prince Sattam bin Abdulaziz University, Al-Kharj, Saudi Arabia.

**Keywords:** excimer light, trichloroacetic acid, vitiligo

## Abstract

**Background:**

Vitiligo, a prevalent acquired depigmentation condition, involves a progressive melanocyte loss, posing significant treatment challenges. Excimer light has been employed in vitiligo treatment, while trichloroacetic acid (TCA) has been noted to induce chemical trauma, leading to perifollicular pigmentation and perilesional repigmentation in vitiligo. This study aims to assess and compare the efficacy and safety of using 308 nm monochromatic excimer light alone versus combining it with 50% TCA in treating vitiligo.

**Methods:**

A total of 50 patients diagnosed with stable vitiligo were included. Each patient had 2 patches of vitiligo selected, with 1 patch treated using 50% TCA and excimer light, while the other patch received excimer light treatment alone.

**Results:**

All patients experienced a notable decrease in the surface area of their vitiligo lesions. However, those treated with excimer light combined with TCA exhibited a greater reduction in lesion size and an increased number of patients showing significant repigmentation compared to lesions treated with excimer light alone. Additionally, patients expressed a higher level of satisfaction with the patches treated with excimer light and TCA compared to those treated with excimer light alone.

**Conclusion:**

The combined treatment of excimer light and TCA shows promise as an effective and safe therapy for vitiligo.

## 1. Introduction

Vitiligo, a chronic pigmentary disorder, impacts around 1% of the global population, with prevalence rates ranging from 0.1% to over 8.8% across different regions.^[1]^ This condition manifests as depigmented patches on the skin and mucosa due to the selective and progressive depletion of functional melanocytes from the epidermal basal cell layer and hair follicles.^[2]^

The development of vitiligo entails a multifaceted interaction between the extrinsic and intrinsic melanocyte defects, inflammation from the innate immune system, and the destruction of melanocytes mediated by T cells. Treatment goals include not only arresting the advancement of the disease but also encouraging the restoration of pigmentation by facilitating the regeneration, proliferation, and migration of melanocytes.^[3]^

Managing vitiligo poses a considerable challenge, with therapeutic options including topical corticosteroids, calcineurin inhibitors, and various phototherapy modalities such as psoralen plus ultraviolet A, narrowband ultraviolet B, and 308 nm excimer light. Surgical interventions like grafting and transplantation, along with depigmentation treatments and psychological support, may also be considered.^[4]^

Among the non-surgical approaches, phototherapy emerges as a highly effective option, particularly narrow-band UVB, which has shown efficacy in both psoriasis and vitiligo treatment. The 308 nm excimer laser, utilizing xenon and chlorine gases to emit targeted UVB at 308 nm, represents a recent advancement in vitiligo phototherapy.^[5]^ Its coherent pulse emission and tailored parameters offer superior effectiveness and safety compared to traditional narrow-band UVB therapy, potentially stimulating melanocytes in deeper hair follicle reservoirs.^[6]^

Trauma caused by trichloroacetic acid (TCA) chemistry has been noted to induce perifollicular pigmentation in areas with hair. This trauma is known to trigger hyperpigmentation by disrupting the basal cell layer, leading to melanophage accumulation in the upper dermis through pigmentary incontinence. Consequently, this inflammatory reaction stimulates melanin synthesis and its transfer to neighboring keratinocytes, contributing to increased pigmentation.^[7]^ Therefore, our objective was to assess and compare the safety and effectiveness of 308 nm monochromatic excimer light alone versus 308 nm monochromatic excimer light combined with 50% trichloroacetic acid in treating vitiligo.

## 2. Patients and methods

The study protocol was reviewed and approved by the Standing Committee of Bioethics Research (SCBR) at Prince Sattam Bin Abdulaziz University, approval number SCBR-275/2024. All participants provided informed written consent prior to their involvement in the study. The study adhered to Helsinki standards as revised in 2013.

This prospective comparative study enrolled 50 patients diagnosed with stable vitiligo, confirmed through Wood’s light examination. Patients were recruited from the outpatient Dermatology and Andrology clinic at Al-Azhar University Hospital in Assiut between October 2023 and February 2024.

A power calculation was conducted to determine the appropriate sample size. The analysis was based on detecting a clinically significant difference in repigmentation rates between the 2 treatment modalities, with a power of 80% and an alpha level of 0.05. Based on these parameters, a sample size of 50 patients was justified and included in the study. To ensure baseline comparability, the selected vitiligo patches were matched as closely as possible for size, location, and disease severity prior to randomization.

Inclusion criteria encompassed patients of any age or gender with stable vitiligo, while those with active vitiligo lesions, recent vitiligo treatment during the previous 6 months, TCA hypersensitivity, scar or keloid history, active skin infections at the site of treatment, or psychological disorders were excluded. Patients unable to commit to a 3-month follow-up or with unrealistic expectations were also excluded.

All patients underwent a comprehensive history-taking process, which included gathering personal information such as name, age, occupation, and residence. Additionally, their medical background, including the initiation, progression, and duration of vitiligo, as well as any past or family history of vitiligo, premature hair graying, or other medical conditions, was documented. Furthermore, details about any previous treatments, such as phototherapy or oral and topical medications, were recorded.

Furthermore, all patients underwent a thorough general examination and dermatological evaluation to determine the vitiligo type and distribution. This evaluation also aimed to exclude the presence of any other skin conditions, such as the Koebner phenomenon, tendency to form keloids, bleeding disorders, or skin infections.

The treatment assignment for each selected vitiligo patch was randomized using a computer-generated randomization sequence to minimize selection bias. One patch was treated with 50% TCA followed by excimer light therapy, and the other was treated with excimer light therapy alone.

The specified regions were cleaned and sterilized, followed by the gentle application of 50% TCA. The TCA was applied uniformly using a cotton-tipped applicator, with a consistent application time of 1 to 2 minutes per lesion. Neutralization of TCA was achieved by thorough rinsing with water immediately after the designated application time to prevent overtreatment. Additionally, any side effects such as erythema, burning sensation, or discomfort were carefully managed by applying emollients and advising patients to avoid sun exposure in the treated areas. This approach ensured uniformity in TCA administration and minimized the risk of variability in treatment outcomes. After each session, patients were instructed to apply zinc oxide cream twice daily and refrain from removing crusts or peeled skin layers. Sessions were repeated every 2 weeks.

Excimer light sessions for the TCA-treated and untreated patches commenced twice weekly on nonconsecutive days. Initial doses were determined by minimal erythema dose (MED) or set at 100 mJ/cm^2^, with subsequent doses increasing by 20% to 30% per visit to achieve mild erythema, indicating optimal treatment fluency. To ensure the appropriate intensity of excimer light therapy, MED was determined for each patient prior to initiating treatment. A test dose was applied to a small, unaffected area of skin, and the MED was measured by evaluating erythema development 24 hours after exposure. The dose of excimer light therapy was then calibrated individually based on the determined MED to ensure safe and effective treatment tailored to each patient’s skin type and sensitivity.

Digital images were captured for each patient at baseline, weekly before each treatment session, and after the completion of treatment using a Canon Power Shot A3400 IS 16MP digital camera. These images aimed to document the clinical response, including the extent of improvement in pigmentation, recurrence, and any potential side effects. Subsequently, the images were analyzed using Optimas software version 6.2.1, which integrates the photographs with automated digital image analysis to quantify the extent of improvement. To strengthen the study’s objectivity, the Vitiligo Area Scoring Index was employed as a validated scoring system to provide quantitative measurements of repigmentation. Standardized photographic analysis methods were also used to ensure consistency in documenting treatment outcomes.

After 3 months, the effectiveness of the therapy was evaluated by analyzing the level of pigmentation in the treated area utilizing the Physician’s Global Assessment (PGA) scale as follows: no change (0), mild improvement (0–25%), moderate improvement (26–50%), good improvement (51–75%), excellent improvement (>75%). If <25% repigmentation was observed at the end of the 3-month period, it was classified as treatment failure.^[8]^ Additionally, patient satisfaction was assessed using a 5-point Likert scale (1 = very dissatisfied, 5 = very satisfied) to evaluate subjective perceptions of treatment success. The satisfaction ratings were analyzed descriptively and correlated with the repigmentation outcomes to examine their relationship.

Statistical analysis was conducted using Stata/IC version 16.1 for Windows (StataCrop, LLC, College Station). Descriptive statistics were presented in tables as the mean ± SD for quantitative data and as numbers and percentages for qualitative data. Assumptions of normality for continuous variables were tested using the Shapiro–Wilk test, while homogeneity of variance was verified using Levene’s test. Confidence intervals and effect sizes were calculated for key outcomes to enhance the robustness of the findings. To compare the 2 techniques, we employed the unpaired Student *t* test, while the paired *t* test was utilized to compare results before and after treatment within each technique. Variations among categorical variables were investigated through the Chi-square test. A *P* value below .05 was deemed statistically significant.

## 3. Results

In terms of the demographic profile of the participants, the average age was 21.16 ± 12.08 years, ranging from 8 to 57 years. Among the participants, 42 (84%) were female, while the remainder were male. The duration of vitiligo ranged from 1 to 20 years, with a mean duration of 4.86 ± 4.50 years. The focal pattern of vitiligo was observed in 15 (30%) of the participants, whereas 35 patients (70%) exhibited the vulgaris type, as illustrated in Table 1.

**Table 1:** Clinicodemographic data of the studied patients.

Total number	50
Age (yr)	21.16 ± 12.08 [8–57]
Sex	
Female	42 (84.0)
Male	8 (16.0)
Duration	4.86 ± 4.50 [1–20]
Residence	
Rural	28 (56.0)
Urban	22 (44.0)
Family history	
Yes	6 (12.0)
No	39 (78.0)
Pattern	
Vulgaris	35 (70.0)
Focal	15 (30.0)
Site	
Neck	2 (4.0)
Upper extremities	19 (38.0)
Lower extremities	11 (22.0)
Trunk	6 (12.0)
Dorsum of the hand and foot	12 (24.0)

Regarding the clinical comparison of the vitiligo lesion surface area before and after the application of excimer light and TCA, no statistically significant variance was detected in the mean baseline surface area of the lesions treated with excimer light plus TCA compared to those treated with excimer light alone. However, patients gave a higher rating and level of satisfaction with the lesions treated with excimer light plus TCA compared to those treated with excimer light alone, with a highly significant *P* value of .001, as shown in Table 2.

**Table 2:** Comparison of mean values of surface area reduction in excimer light plus TCA versus excimer light alone.

	Excimer + TCA	Excimer alone	*P* value
Surface area (cm^2^)			
Before	9.30 ± 13.24	10.37 ± 14.54	.7004
After	1.65 ± 3.85	5.36 ± 8.44	.0057*
Mean difference	7.64 ± 10.59	5.01 ± 8.89	.0053*
Degree of satisfaction			
Very satisfied	33 (66.0)	7 (14.0)	<.001*
Satisfied	17 (34.0)	36 (72.0)	
Poorly satisfied	0 (0.0)	7 (14.0)	

TCA = trichloroacetic acid.

* No statistically significant variance was detected in the mean baseline surface area of the lesions treated with excimer light plus TCA compared to those treated with excimer light alone. However, patients gave a higher rating and level of satisfaction with the lesions treated with excimer light plus TCA compared to those treated with excimer light alone, with a highly significant *P* value of .001.

**Figure 1.**
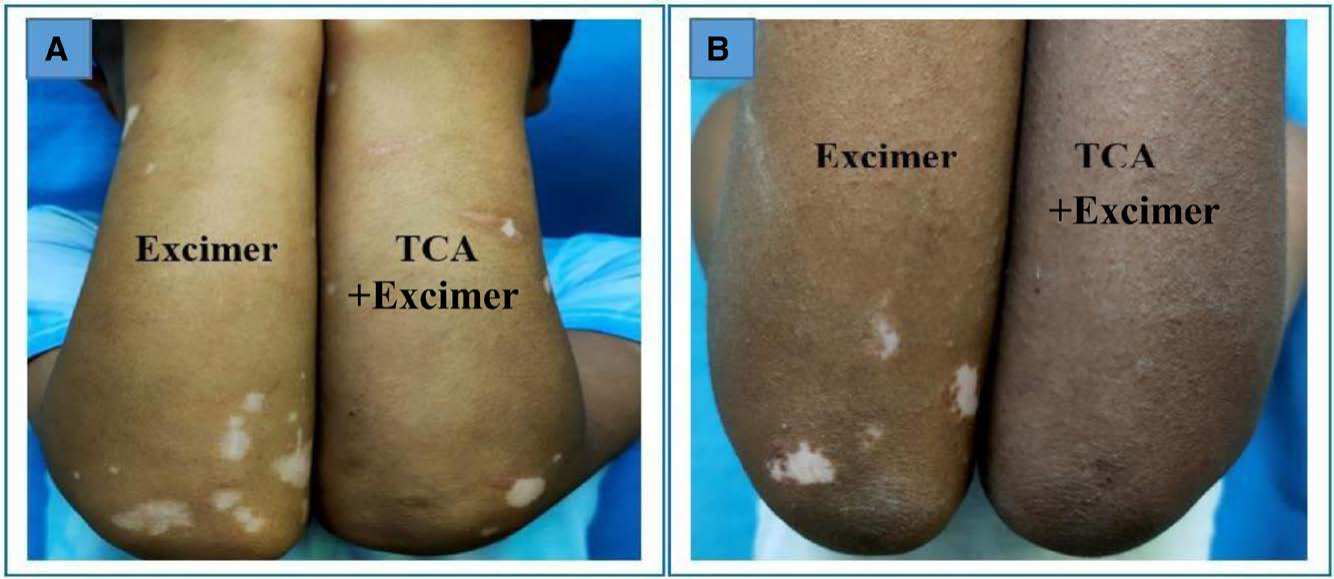
Male patient 12 years old complaining of stable vitiligo vulgaris on both forearms of 2 years duration, (A) before and (B) after 3 months of treatment (left side treated with 50% TCA plus excimer light & right side treated with excimer light alone), with marked pigmentation noted in TCA treated side. TCA = trichloroacetic acid.

**Figure 2.**
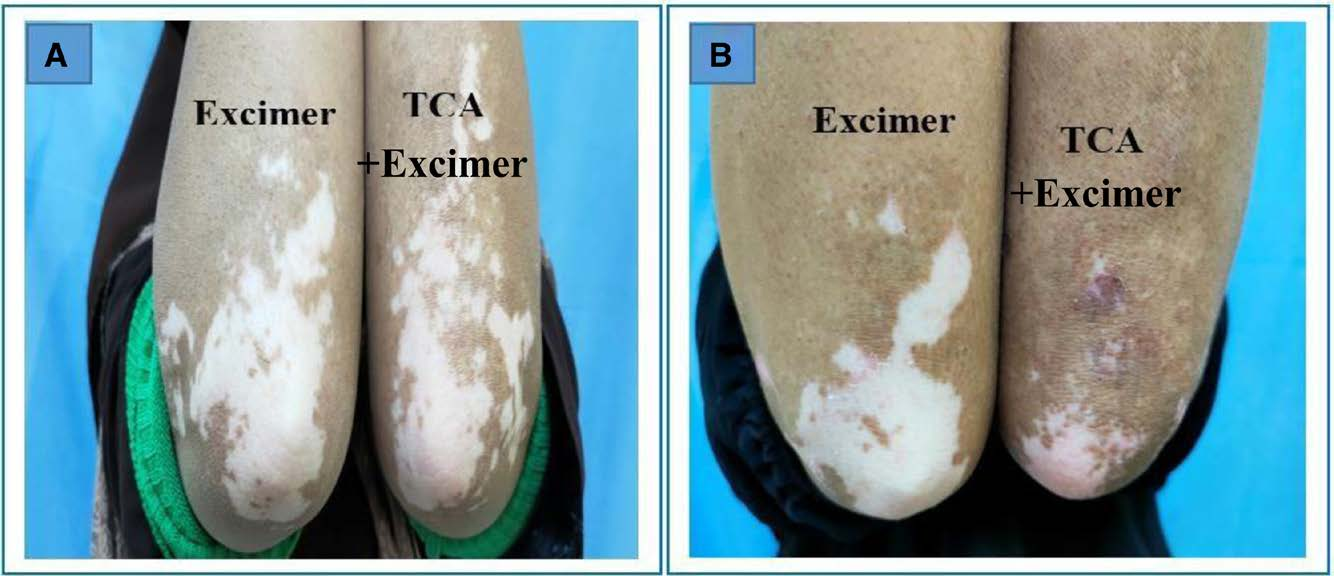
Female patient 30 years old complaining of stable vitiligo vulgaris on both forearms of 2 years duration, (A) before and (B) after 3 months of treatment (left side treated with 50% TCA plus excimer light & right side treated with excimer light alone), with marked pigmentation noted in TCA treated side. TCA = trichloroacetic acid.

**Figure 3.**
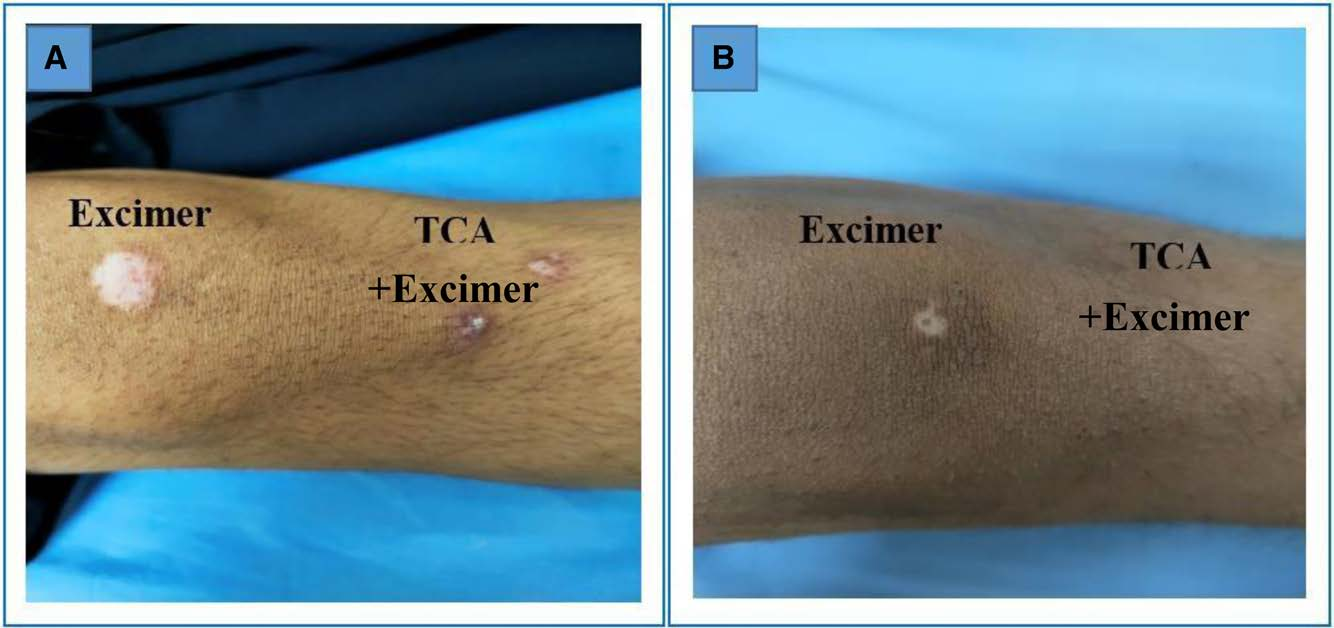
Male patient 11 years old complaining of focal stable vitiligo on left knee of 7 years duration, (A) before and (B) after 3 months of treatment (the left patch treated with excimer light alone & the right patch treated with 50% TCA plus excimer light), with marked pigmentation noted in TCA treated side. TCA = trichloroacetic acid.

**Figure 4.**
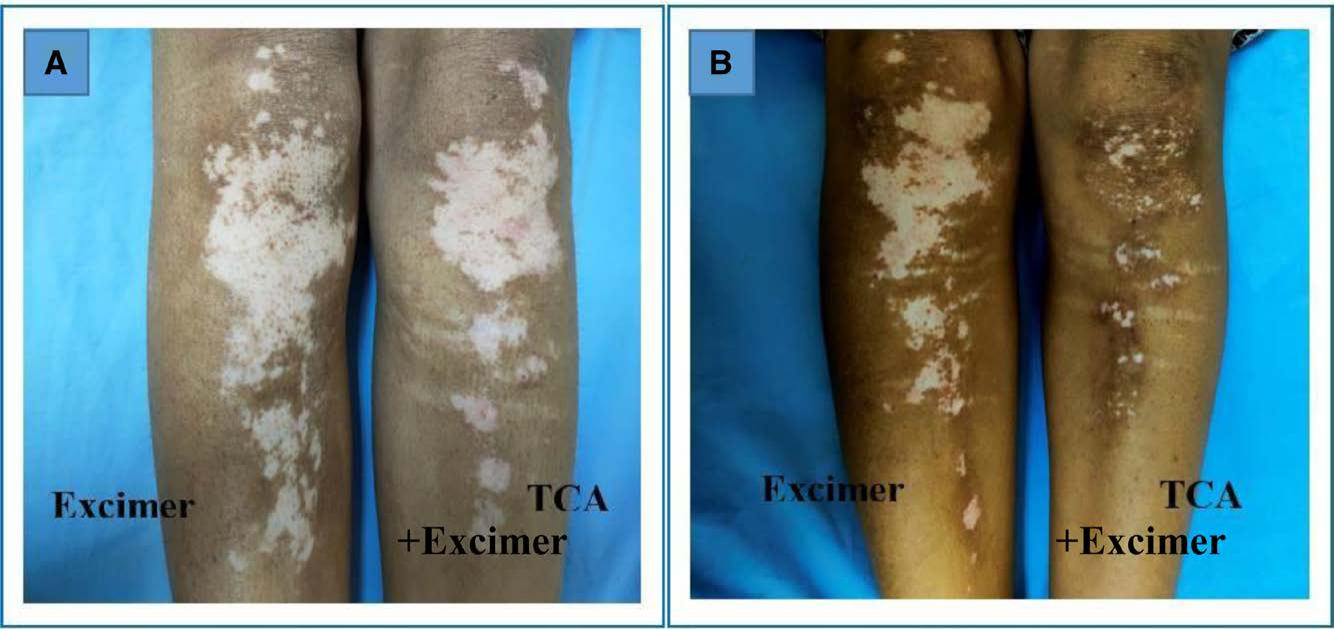
Female patient 30 years old complaining of stable vitiligo vulgaris on both legs of 10 years duration, (A) before and (B) after 3 months of treatment (left side treated with 50% TCA plus excimer light & right side treated with excimer light alone), with marked pigmentation noted in TCA treated side. TCA = trichloroacetic acid.

**Figure 5.**
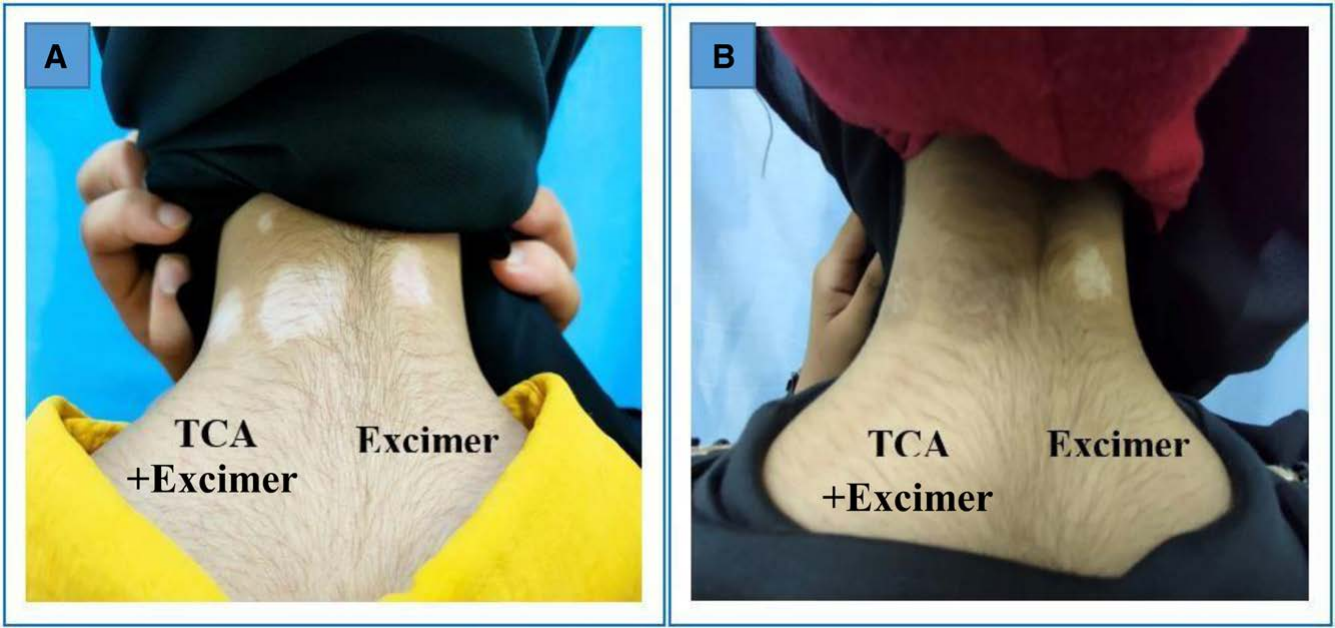
Female patient 23 years old complaining of stable focal vitiligo on the nape of the neck of 4 years duration, (A) before and (B) after 3 months of treatment (2 patches on right half treated with 50% TCA plus excimer light & the patch on left half treated with excimer light alone), with marked pigmentation noted in TCA treated side. TCA = trichloroacetic acid.

Regarding the repigmentation of skin lesions, significant differences were observed in the degree of improvement between the group that underwent combination therapy and those treated with excimer light alone (Figs. 1–5), with a highly significant *P* value of <.001. However, there was no significant difference in the pattern of repigmentation between the lesions treated with excimer light plus TCA and those treated with excimer light alone, as illustrated in Table 3.

**Table 3:** Comparison between excimer light plus TCA versus excimer alone according to degree and pattern of repigmentation.

Degree of repigmentation	Excimer + TCA	Excimer	*P* value
Marked repigmentation > 75%	33 (66.0)	9 (18.0)	<.001*
Moderate repigmentation 50–75%	13 (26.0)	19 (38.0)	
Mild repigmentation 25–49%	4 (8.0)	14 (28.0)	
Treatment failure; repigmentation < 25	0 (0.0)	8 (16.0)	

Pattern of regimentation			
Peripheral	33 (66.0)	9 (18.0)	.369
Perifollicular	13 (26.0)	19 (38.0)	
Mixed	4 (8.0)	14 (28.0)	

TCA = trichloroacetic acid.

* No statistically significant variance was detected in the mean baseline surface area of the lesions treated with excimer light plus TCA compared to those treated with excimer light alone. However, patients gave a higher rating and level of satisfaction with the lesions treated with excimer light plus TCA compared to those treated with excimer light alone, with a highly significant *P* value of .001.

Referring to the negative outcomes of the procedures, the majority of patients undergoing both techniques experienced no complications. Nonetheless, instances of erythema, burning, and irritation were more prevalent on the side treated with excimer plus TCA, with no notable disparity between the 2, as demonstrated in Table 4.

**Table 4:** Complication of excimer plus TCA versus excimer alone.

Complication	Excimer + TCA	Excimer alone	*P* value
No complication	38 (76.0)	45 (90.0)	.152
Erythema	8 (16.0)	5 (10.0)	
Burning	3 (6.0)	0 (0.0)	
Irritation	1 (2.0)	0 (0.0)	

TCA = trichloroacetic acid.

## 4. Discussion

Vitiligo, a common manifestation of acquired skin hypomelanosis, poses a significant therapeutic challenge due to the chronic and unpredictable nature of its course. The condition, characterized by distinct depigmented macules and patches often accompanied by leukotrichia, requires comprehensive management strategies aimed at restoring pigmentation to affected areas.^[9]^ However, despite the array of treatment modalities available, many of them entail prolonged treatment durations and offer only marginal efficacy.^[10]^

Among the diverse therapeutic approaches, light-based interventions have garnered considerable attention, particularly 308-nm excimer light therapy. This modality has shown promise in delivering significant repigmentation outcomes within a condensed temporal framework.^[11]^ Additionally, TCA has emerged as a straightforward option for vitiligo treatment, yielding cosmetically satisfactory repigmentation results. Moreover, TCA can be employed preemptively or concurrently with established vitiligo treatment modalities, further enhancing its versatility in clinical practice.^[12]^

This investigation aimed to evaluate the efficacy and safety of 308-nm monochromatic excimer light monotherapy versus a combined regimen of 308-nm excimer light and 50% TCA for vitiligo management. We observed a notable reduction in the mean baseline surface area of vitiligo lesions across all patients treated with both techniques. Moreover, a higher proportion of patients exhibited marked repigmentation when treated with the combined regimen (Figs. 1–5), indicating its potential superiority over excimer light monotherapy. Additionally, patients treated with the combined approach reported higher levels of satisfaction.

The synergistic effects of TCA and excimer light therapy observed in this study could be attributed to complementary mechanisms of action. TCA-induced chemical trauma likely triggers a localized inflammatory response, promoting the release of growth factors such as endothelial and fibroblast growth factors. These factors are known to stimulate melanogenesis, enhance melanocyte migration, and initiate wound-healing processes that contribute to repigmentation. Furthermore, excimer light therapy stimulates keratinocyte and fibroblast proliferation, enhancing their interaction with melanocytes to foster repigmentation.^[13,14]^

The potential role of TCA in modifying the inflammatory microenvironment deserves further exploration. By inducing controlled epidermal peeling, TCA may enhance the accessibility of melanocytes to treatment modalities like excimer light, thereby amplifying its effects. Additionally, the oxidative stress generated by TCA might stimulate melanocyte activity and migration from adjacent healthy skin or hair follicles.^[14]^

Our findings align with previous studies demonstrating the efficacy of TCA as a standalone or combined therapy.^[15–17]^ Ibrahim et al^[17]^ reported good to excellent repigmentation (>50%) using 70% TCA with microneedling, while our study achieved even higher rates (>75%) with the addition of excimer light therapy. This suggests that the enhanced repigmentation in our study may be attributed to the synergistic effects of combining these 2 treatment modalities.

Other studies utilizing TCA in conjunction with narrowband ultraviolet B^[18]^ or microneedling^[19]^ have reported varied repigmentation outcomes. Notably, Elnokaly et al^[19]^ achieved 43.3% repigmentation with TCA 25% combined with tacrolimus and microneedling, whereas our higher concentration of TCA (50%) coupled with excimer light therapy yielded superior results. This discrepancy underscores the potential impact of TCA concentration and adjunctive therapies on treatment efficacy.

El Mofty et al^[20]^ found that TCA 25% was the most effective modality among their tested therapies, achieving perifollicular pigmentation and marginal tanning. Our findings corroborate these results, further emphasizing the efficacy of TCA in stimulating melanocyte activity when combined with advanced phototherapy.

Additionally, Yang et al^[21]^ demonstrated enhanced repigmentation with combined excimer light therapy and electrocautery needling. Their findings highlight the potential of excimer light therapy to amplify the effects of adjunctive techniques, consistent with our observations.

Studies on excimer light therapy alone have shown varying degrees of repigmentation.^[21,22]^ Fa et al^[22]^ reported repigmentation exceeding 76% in 34% of patients, which aligns with our findings of higher repigmentation rates (>75%) in the combined therapy group. These results underscore the efficacy of excimer light therapy, especially when augmented by adjunctive treatments like TCA.

Both excimer light therapy and TCA demonstrated tolerable safety profiles in our study, with minor adverse effects such as erythema, burning sensations, and irritation. These effects were transient and manageable, even with the combined therapy. Literature supports the safety of excimer light therapy, with occasional photosensitivity and hyperpigmentation.^[21–23]^ Similarly, TCA-associated adverse effects are well-documented as mild and temporary.^[15,19]^

In conclusion, the combined use of 308-nm excimer light and 50% TCA offers a promising therapeutic approach for vitiligo management, achieving superior repigmentation outcomes compared to monotherapy. The mechanistic synergy between TCA-induced chemical trauma and excimer light-mediated melanocyte stimulation provides a compelling basis for further investigations into optimized combination regimens.

## 5. Conclusion

Our study contributes to the growing body of evidence supporting the combined use of excimer light and TCA for vitiligo management. The findings underscore the potential synergistic effects of these modalities in enhancing repigmentation outcomes while maintaining satisfactory safety profiles. Further research is warranted to explore optimal concentrations and application protocols to maximize therapeutic outcomes while minimizing adverse effects.

## 6. Limitations

Our study has several limitations. Firstly, we exclusively recruited patients from the Upper Egypt area, highlighting the potential influence of geographical and ethnic backgrounds on the clinical manifestations of pigmented skin disorders. Secondly, the small sample size and relatively short study duration may lead to notable differences between our findings and those of other studies. Additionally, the short follow-up duration limits our ability to assess the long-term efficacy and safety of the combined treatment regimen. Future studies should aim to include extended follow-up periods to provide more robust data on sustained outcomes. Another significant limitation is the absence of long-term safety data. Although both treatment modalities demonstrated tolerable safety profiles within the study period, further research is needed to evaluate potential delayed adverse effects, especially when using higher concentrations of TCA. Moreover, the study lacked blinding, which could introduce bias into patient-reported outcomes and the assessment of treatment efficacy. Employing double-blind designs in future investigations would help mitigate this limitation and enhance the reliability of findings. Finally, the potential for a placebo effect cannot be excluded, as the visible peeling effect of TCA might influence patients’ perception of treatment efficacy. Incorporating a control group receiving placebo interventions would provide a more robust framework for evaluating the true therapeutic benefit of the combined regimen. Addressing these limitations in future research will strengthen the evidence base for the combined use of TCA and excimer light therapy in vitiligo management.

## 7. Recommendations

We advocate for the utilization of a combination therapy involving TCA and excimer light in the treatment of vitiligo, owing to its safety, simplicity, tolerability, and cost-effectiveness. Additionally, we recommend validating our findings through additional studies.

## Author contributions

**Conceptualization:** Ramadan S. Hussein.

**Data curation:** Salman Bin Dayel, Refaat R. M. Hammad.

**Formal analysis:** Salman Bin Dayel, Refaat R. M. Hammad.

**Investigation:** Ramadan S. Hussein, Refaat R. M. Hammad, Othman Abahussein.

**Methodology:** Ramadan S. Hussein, Shaimaa E. A. Badawy.

**Resources:** Salman Bin Dayel, Shaimaa E. A. Badawy, Mofreh Mansour.

**Supervision:** Othman Abahussein.

**Validation:** Abeer Ali El-Sherbiny, Mofreh Mansour.

**Visualization:** Refaat R. M. Hammad, Othman Abahussein.

**Writing – original draft:** Shaimaa E. A. Badawy.

**Writing – review & editing:** Abeer Ali El-Sherbiny, Mofreh Mansour.
